# Differentiation of Typhoid and Paratyphoid A and B Bacilli by a Dextrin-inosite Medium

**Published:** 1919

**Authors:** 


***Differentiation of Typhoid and Paratyphoid A and B Bacilli by a Dextrin-inosite Medium.*** By FLORENCE HULTON-FRANKEL. *Journal of Infectious Diseases*, October 1918.

The medium was an ordinary 3 o/o infusion agar with usual typhoid reaction of about 0.7 acid. One per cent. of both dextrin and inosite were added; dextrin used was Merck's C. P.; inosite was made in the laboratory by Nelson's method.

The results showed :

The typhoid bacillus ferments dextrin-inosite with acid formation.

The paratyphoid bacillus B ferments dextrin-inosite medium with acid and gas formation.

The paratyphoid bacillus A does not ferment dextrin-inosite.

The dysentery group falls into two groups; the Shiga-Bruss and

Hiss-Russel types do not ferment dextrin-inosite, while the Flexner-Rosen types do.

B. typhi murium and B. pullorum do not ferment dextrin-inosite.

B. aerogenes ferments dextrin-inosite with gas formation.

The dextrin-inosite medium differentiates between the typhoid bacillus and paratyphoid bacilli A and B, although it does not differentiate between the paratyphoids and the colon bacilli.

In conjunction with the Russel double sugar agar it gives confirmatory evidence of the identity of the typhoid bacillus and distinguishes between the paratyphoid bacilli A and B.

